# Is It Time for Quotas to Achieve Racial and Ethnic Representation in Multiple Sclerosis Trials?

**DOI:** 10.3389/fneur.2021.680912

**Published:** 2021-05-13

**Authors:** Farrah J. Mateen

**Affiliations:** Department of Neurology, Harvard Medical School, Massachusetts General Hospital, Boston, MA, United States

**Keywords:** multiple sclerosis, diversity, race, ethnicity, socioeconomic status, USA, clinical trial, quota

Clinical trials form the bedrock of evidence upon which clinical decision making is based. In multiple sclerosis (MS), the number of disease modifying therapies (DMTs) continues to expand with the number of clinical trials growing rapidly, especially over the past two decades. Phase III trials of DMTs are usually multi-centered, spanning many countries, and involve dozens of centers and hundreds of investigators. Meanwhile, efforts to improve racial and ethnic diversity in clinical trials in general in the United States have largely failed (1, 2). Just 5% of trial participants are African American or Black in the USA (compared to 13% of the population in the last reported U.S. census) and just 1% are Hispanic/LatinX (compared to 18% of the U.S. population) (2). Clinical trials for people living with MS are no exception to this reality (3, 4).

Current efforts to advance health equity via phase III clinical trials in MS appear inadequate. MS research is highly sensitive to the many structural inequities experienced across the U.S.A. Although clinical trials in MS have often been considered separate from broader social, cultural, and political trends, demands for equity in all aspects of American society are rightly becoming a *tour de force*. Many stakeholders are beginning to re-examine who enters and leads clinical trials. Increasing scrutiny of the representativeness of people of color is therefore rightly gathering new attention by patients, investigators, and the general public as the science of MS rapidly advances.

Quotas, or enrollment number targets, are one approach that may be taken to increase the number of people of color in MS clinical trials. This practice has recent precedents. Taking a sense of responsibility for racial and ethnic representativeness to a new level, one recent trial of a vaccine for SARS-CoV-2 by a U.S.-based company set targets for the enrollment of participants from specific racial and ethnic groups in the U.S. and slowed enrollment to ensure adequate representation. Others have followed this trend by publicly delaying completion of enrollment in their trial until benchmarks for representativeness are met (5, 6). These benchmarks approximate the incidence of the disease in the target population for the proposed product and recognize concentrated epidemics within epidemics. In other sectors, most prominently including corporate America, the role of target numbers or quotas for the inclusion of people of color and women on executive boards has become a matter of not only public trust and confidence, but also enforced regulation. In 2020, California *bill AB 979* was passed to eliminate all-White boards of publicly traded companies headquartered in that state (7). Accountability by major companies to achieve representativeness is considered a public good and sets a new standard which clinical trials can learn from.

There are reasons for and against concrete numerical enrollment targets to ensure representativeness of people of color in MS clinical trials (Table 1).

**Table 1:** Summary of arguments *For* and *Against* enrollment targets (quotas) to improve racial and ethnic diversity in MS late-phase therapeutic clinical trials.

**For**	**Against**
Reflect the true epidemiology of the disease across races and ethnicities	Lack of interest, willingness, trust among people who are targeted for enrollment
Explore differences and include variations in putative disease risk factors	Trial does not advance the field enough, generating limited enthusiasm for enrollment
Understand any differences in therapeutic response	The pace of science slows for all people with the disease
Adequately power subgroup analyses in regards to race and ethnicity, even if through meta-analyses	Targets reflect minimum standards but not maximal progress on larger issues of health equity
Equitably distribute possible benefits to trial participants, including earlier access to improved therapies	Race is a social construct that does not adequately reflect genetic diversity
Recognize that passive efforts to achieve diversity and representativeness in clinical trials have fallen short	Increased costs to performing the trial which may limit the trial in other important aspects, e.g. duration, sample size, biomarkers
Avoid post-trial smaller studies focused on ethnic and racial groups after pivotal trials are complete	Ability of participants to access DMTs and health services following trial discontinuation is not guaranteed
Meet societal expectations for representation in all sectors and aspects of society	Negative perceptions by communities of color; efforts may backfire
Investment in broader aspects of infrastructure and processes during trial performance that ultimately improve health equity for more people with MS, including future patients and participants	Other efforts to improve health equity and diversity may be preferable, even if not yet done

First among the reasons for enrollment targets, there is evidence that MS may differ in both risk factors and disease severity in certain racial and ethnic groups. People who identify as African American, LatinX, Middle Eastern, and North African are reported to have increased disease activity, more lasting disability, and/or worse clinical outcomes (8–13). Other characteristics relevant to MS including early life exposures or vitamin D levels may differ (14). The predilection for spinal cord disease with its resultant disability may be higher in African Americans compared to Caucasian Americans (9), although this is debated. Response to DMTs in certain groups may or may not differ. Since subgroup analyses are not statistically powerful enough to answer concerns around DMT responsiveness in different racial and ethnic groups, even meta-analysis across phase III clinical trials cannot definitively answer questions on racial and ethnic differences in DMT efficacy in 2020 (4). This is in spite of several thousands of MS participants over an entire generation of research. Indeed, some high profile randomized clinical trials in MS do not even report on race and ethnicity in their published manuscripts, likely concealing the low enrollment of people of color in these otherwise pivotal studies.

Second, benefits may occur to participants in clinical trials. Many people with MS in clinical trials have gained earlier access to higher efficacy therapies, often at no cost, and have been rightfully paid to participate, including in extension studies over several years (15). Since clinical trials are concentrated in academic, usually urbanized centers, they tend to enroll participants who have access to specialized MS care. These centers, even if located in cities of highly diverse racial representation, may not actually serve the most disadvantaged populations in their outpatient MS clinics. Enrollment of participants from investigators' own clinics is usual and cost-effective but is not always the most diversifying strategy. According to the Zip Code Analysis Project in the U.S.A., 80% of U.S. people who identify as minorities live in 20% of the zip codes (2). Access to people from these 20% of zip codes vs. clinical trial enrollment sites is yet to be well-analyzed.

Third, passive or even encouraging and energetic efforts to improve racial and ethnic diversity in clinical trials have not led to broad representation to date. Since these efforts are not generally tracked or reported alongside MS trial results, the degree to which concerted efforts have been made across trial planners to optimize representativeness is unclear. Phase III trials for some of the earliest approved MS DMTs, beginning in the early 1990s, had at most 7% enrollment by African Americans with 93–98% of participants reported as white across trials (3). The four most recent, newly-approved novel drugs by the U.S. Food and Drug Administration (FDA) are reported by participant characteristics via the Center for Drug Evaluation and Research. In their “Drug Trials Snapshots,” the percentages of people who are Black, Hispanic, or Asian are overall low: Zinbryta (1% Black, 3% Hispanic, not reported for Asian, approved in 2016); Ocrevus (4, 0, 11%, 2017); Mayzent (1, 3, 6%, 2019); and Zeposia (1, 0, 1%, 2020) (16). After several decades, merely stating interest in under-represented groups in trial enrollment has therefore not progressed to representative trial enrollment. It must also be noted that enrollment in these trials occurs mostly outside of the USA, with no more than 26% of enrolled participants coming from the USA among the three most recently reported FDA approvals for novel MS DMTs (16). Since most calls for diversity have come from public funders, such as the U.S. National Institutes of Health, but most phase III trials are now funded through pharmaceutical and private sources, efforts by U.S. government funders alone are insufficient to make the strides needed to fully diversify MS clinical trials research. Although racial and ethnic diversity may be part of a broad interest of trial planners, pressure to include racially and ethnically diverse populations may be much less than other pressures to bring a new drug successfully to market.

Groups that are traditionally called “under-represented” are under-represented in clinical trials for important reasons, including structural racism and socioeconomic disadvantage that may be colinear with race and ethnicity in some situations. To engage those of socioeconomic disadvantage requires often increased—although certainly not insurmountable—research costs such as inclusion of additional study sites, increased transportation assistance for participants' visits, coverage of care for dependents during trial participation, and materials that are accessible to people across the so-called digital divide. As just one example, still approximately 20 million Americans lack access to broadband connections in the U.S. (17). It is likely that many people of socioeconomic disadvantage and certain groups who experience the digital divide in the USA—such as many U.S. Native Americans (or Indigenous Americans) and Alaskan Natives—are not as actively seeking trials online via clinicaltrials.gov or able to access online information as readily. Other efforts may include translation of recruitment and study documents to multiple other languages to improve access to people with MS across multiple languages.

There may similarly be many reasons not to set targets or quotas for race and ethnicity in MS clinical trials. People of color with MS may have less interest, trust, or willingness to engage in clinical trials in some situations, because the trial appears risky, lacks promise, or does not meet their needs. There are historical examples why this mistrust of trials exists and should be taken seriously (18). Since an increasing number of people with MS may be stable on DMTs, and some trials are non-inferiority studies of biosimilar products, it is clear that each trial is not created equally, and each trial does not necessarily advance the field to the same degree. An argument could be made that enrollment targets ultimately slow down the pace of science and delay progress in drug development, a situation that potentially helps no one. Targets, according to some leaders in America, do not reflect success in representativeness, but overt failures, since all other more voluntary mechanisms to improve diversity have to date therefore failed. Targets may thus reflect minimum standards but not maximal progress.

Agreement on what a target is overall, including whether that target should reflect the disease epidemiology by race and ethnicity, the U.S. population in general, or a more global metric remains unclear and could become highly controversial. Moreover, enrollment is only one metric of a study's success. Other aspects of trial participation, including study completion metrics may be preferable. Similarly, other disease or demographic factors may be universally more important to predicate a study's enrollment upon in order to answer the primary study question.

Since the epidemiology of MS in the USA is modeled, usually based on medical billing data, the exact numbers of people living with MS who identify with different races and ethnicities are still somewhat unknown. The categorization of race and ethnicity may include people who self-identify with many backgrounds. Categories for race and ethnicity are not agreed upon and differ across reports. Self-report may be required but may dissuade participation of some people with MS in clinical trials that are trying to recruit them. Race is a social construct, a poorly defined marker of genetic diversity, and an imprecise proxy for the relationship between genetics and ancestry (19). As an extreme situation, race as a reason to use or not use a specific MS drug would be an unintended consequence of such efforts.

An additional major consideration is cost and whether increased costs to recruit and enroll more difficult to reach participants will still lead to adequate powering of subgroups of interest to answer fundamental biological questions. Continuing access to DMTs after a trial is not a guarantee for participants, particularly for the many years if not decades that the DMT could be used after a trial. If participants' health insurance or public payor coverage fails to continue the DMT they were given during the trial, additional problems could arise for the participant after trial completion. These include both clinical concerns such as “rebound” after immunosuppression changes and socioeconomic concerns such as untenable copay coverage.

Since the primary underlying goal of enrollment targets is to improve health equity, enrollment targets are simply a concrete, if not superficial, way to measure and mandate progress toward a much larger issue. The root factors that led to a lack of diversity and representativeness in MS clinical trials deserve dedicated study since this literature is extremely sparse for people with MS. In other disorders, initiatives have included multi-sector action plans, dedicated patient focus groups, large qualitative studies, and mixed methods designs to understand the true causes of the underlying issues (20–22). The perception of communities of color should be specifically considered when deciding whether or not to implement enrollment targets since such major steps are not without potential risks.

Direct efforts to understand non-participation could also challenge false assumptions (23), such as lack of interest or knowledge about MS trials among certain racial and ethnic groups. Factors reported in other medical conditions include uncertainty of participants on their safety, inadequate communication on research opportunities and findings, and lack dissemination of information from active trials (20). Efforts by trial organizers to build community trust, achieve cultural competence and humility (20, 21), consider participants' comorbidities, and prevent dropouts should be prioritized (22).

Diversity and representativeness are favorable, necessary, and overdue in the field of MS. Realization of representativeness requires decision making. Concrete recommendations to achieve what may seem to be lofty goals include: mandatory reporting of race and ethnicity in all MS clinical trials; an online report card for trials on the issue of racial and ethnic diversity but also the very important drivers of outcomes including social determinants of health; shared commitments among new and established MS DMT manufacturers to improve representativeness of people of color in their pivotal studies rather than *post-hoc* analyses and add-on trials years afterwards; and improving the diversity and representativeness of the investigators, study staff, and public communicators of the final study findings. Building trust and confidence among all people with MS, present and future, is an important motivation and a potential result of doing better. Translating research into public action and harnessing societal expectations for MS clinical trials is timely and necessary. Enrollment targets have risks but, at least in the short term, are a singularly clear mechanism to remain accountable and attempt to ensure research progress is collective.

## Author Contributions

FM contributed to the conception and design of the work, drafting, and critical revision of the article, and final approval of the version to be published.

## Conflict of Interest

FM has research funding from the National Institutes of Health and IQVIA (EMD Serono, Genentech) and prior research funding from the National MS Society and Biogen, unrelated to this topic. She has received consulting fees from Biogen and Genentech, unrelated to this topic.
